# Microbiota activation and regulation of adaptive immunity

**DOI:** 10.3389/fimmu.2024.1429436

**Published:** 2024-10-09

**Authors:** Mozhdeh Heidari, Saman Maleki Vareki, Ramin Yaghobi, Mohammad Hossein Karimi

**Affiliations:** ^1^ Transplant Research Center, Shiraz University of Medical Sciences, Shiraz, Iran; ^2^ Department of Oncology, Western University, London, ON, Canada; ^3^ Department of Pathology and Laboratory Medicine, Western University, London, ON, Canada; ^4^ London Regional Cancer Program, Lawson Health Research Institute, London, ON, Canada

**Keywords:** microbiota, adaptive Immunity, T cells, B cells, regulatory T cells

## Abstract

In the mucosa, T cells and B cells of the immune system are essential for maintaining immune homeostasis by suppressing reactions to harmless antigens and upholding the integrity of intestinal mucosal barrier functions. Host immunity and homeostasis are regulated by metabolites produced by the gut microbiota, which has developed through the long-term coevolution of the host and the gut biome. This is achieved by the immunological system’s tolerance for symbiote microbiota, and its ability to generate a proinflammatory response against invasive organisms. The imbalance of the intestinal immune system with commensal organisms is causing a disturbance in the homeostasis of the gut microbiome. The lack of balance results in microbiota dysbiosis, the weakened integrity of the gut barrier, and the development of inflammatory immune reactions toward symbiotic organisms. Researchers may uncover potential therapeutic targets for preventing or regulating inflammatory diseases by understanding the interactions between adaptive immunity and the microbiota. This discussion will explore the connection between adaptive immunity and microbiota.

## 1 Introduction

The human body is colonized by trillions of microbes, which collectively form a microbial community known as the human microbiome. The original discovery of “microbiota” dates back to the early 1900s. It was found that a vast number of microorganisms, consisting of viruses, yeasts, and bacteria coexist in various sites of the human body (the gut, skin, lung, and oral cavity) (1–3). A large percentage colonizes the gastrointestinal (GI) tract, which is called the gut microbiota (4, 5). Gut microbiota plays an essential role in the host’s metabolism and immunity. The gut microbiota metabolizes proteins and complex carbohydrates, synthesizes vitamins, and produces many metabolic products, mediating the crosstalk between the gut epithelial and immune cells (6). The gut microbiota significantly influences the development of host immunity. Conversely, the microbiota is regulated by the immune system through the maintenance of the intestinal barrier. These interactions are typically homeostatic, carefully controlled, and governed by innate and adaptive immune responses (7–9). The microbiota imbalances, referred to as “dysbiosis, “ have been associated with a multitude of diseases of various etiologies. This includes inflammatory bowel disease, autoimmunity, metabolic syndrome, and even neurodevelopmental disorders (10). One of the major mechanisms by which the microbiota has been demonstrated to impact these diseases is through its chronic interactions with and impact on the host immune system. Various microbiota derivatives and metabolites can affect the host’s intestinal immune system by altering the behavior of diverse cell types. Intestinal epithelial cells (IECs), mononuclear phagocytes, innate lymphoid cells (ILCs), and B and T lymphocytes are among the cell types found in this system (11). The most abundant microbiota-derived metabolites in the gut lumen include dietary fiber and short-chain fatty acids (SCFAs). They primarily play critical roles in inflammatory signaling, protecting against pathogen invasion, and maintaining barrier integrity (12, 13). SCFAs are also crucial regulators of immune cell activation, recruitment, and differentiation, including neutrophils, macrophages, dendritic cells (DCs), and T-lymphocytes (14).

Commensal microbiota initiates both innate and adaptive intestinal immune responses, coordinating host protection from pathogens and intestinal homeostasis (15, 16). The innate immune system rapidly responds to the gut microbiota in a nonspecific antigen manner through the activation of pattern recognition receptors. It releases cytokines such as interferon-α, interleukin-18 (IL-18), and interleukin-22 (IL-22) to promote epithelial antimicrobial responses, namely the production of antimicrobial peptides (17).

The intestinal resident microbiota’s influence on the intestinal adaptive immune response involves the differentiation of CD4+ T cells, IgA-producing B cells in Peyer’s patches(PPs), and cells in the lamina propria. Intestinal epithelial lymphocytes (IELs) are essential for immune tolerance towards symbiotic bacteria, the intestine barrier’s integrity, and gut homeostasis (18). The adaptive immune system identifies distinct microbial antigens via its highly variable surface receptors (19). Naive T cells can transform into effector T cells or regulatory T cells Tregs, depending on the type of bacteria they encounter. Although it takes time for the adaptive immune system to differentiate and proliferate to respond to microbial antigens after the first encounter, some antigen-experienced memory cells exhibit long-term survival and provide a robust and timely response in a recall encounter (20). This review focuses on the interaction between the host immune system and gut microbiota to explore the role of the adaptive immune system in establishing symbiotic relationships with the gut microbiota.

## 2 The adaptive immune system and microbiota diversity

In the human body, the adaptive immune system intricately collaborates with microbiota. Adaptive immunity can detect and respond effectively to any pathogen or transformed cellular antigen. The adaptive immune system consists of various types of T cells and B cells. These cells ensure proper immune function by suppressing responses to non-harmful antigens and protecting the gut mucosa’s barrier functions, thereby playing an essential role in maintaining immune homeostasis. During stable conditions, gut microbiota and intestinal T cells interact harmoniously to influence the overall immune system response. A system of checks and balances between potentially proinflammatory cells is responsible for the homeostasis in the gut mucosa. This system is including Th1 cells that produce IFN-γ; TH17 cells that produce IL-17A, IL-17F, and IL-22; TH2; and anti-inflammatory Foxp3+ regulatory T cells (T-regs) (21, 22).

By producing large amounts of secretory immunoglobulin A(sIgA) that respond to common bacteria, B cells can maintain intestinal homeostasis. Secretory IgA can be produced through both T cell-independent and T cell-dependent processes. To shape the microbial community in the gut, IgA production via a T cell-dependent pathway is essential. These sIgAs coat commensal bacteria with their affinity for one another and restrict growth, which prevents them from penetrating the mucosal barrier (23). Furthermore, intestinal innate immunity significantly contributes to the immune-tolerant traits of adaptive immunity (24). Recent studies have shown that interactions between T follicular helper cells and B cells in a steady state are regulated by group 3 innate lymphoid cells, ILC3s, to limit mucosal IgA responses (25). ILC3s promote immune tolerance to the microbiota and protect intestinal health by utilizing antigen-specific RORγt+ Treg cells and Th17 cells.

This immune homeostasis fortifies the host’s defense against pathogens and maintains a balanced immune response toward commensal bacteria (26) (**Table 1**). **Table 1** provides a comprehensive overview of various clinical trials investigating the relationship between gut microbiota and adaptive immunity in different human diseases. For instance, in the study by Cao et al. (2023) on CRC, the authors identified distinct features of TCRβ repertoires in CRC patients compared to healthy donors, suggesting the potential of microbiota-based biomarkers for CRC detection and diagnosis. Similarly, studies on RA by Wang et al. (2022), Li et al. (2020), and Sun et al. (2019) highlight the dysbiosis of certain bacterial lineages and its impact on immune profiles, contributing to the understanding of RA pathogenesis. Moreover, investigations into MS by Siobh_ et al. (2020) and Saresella et al. (2017) reveal associations between gut microbiota diversity, inflammatory T-cell subsets, and disease activity, emphasizing the potential therapeutic implications of modulating gut microbiota composition in MS management. The table also encompasses studies on breast cancer, type 1 diabetes, type 2 diabetes, and CD, elucidating the role of gut microbiota in disease development and progression. For example, Shi et al. (2019) demonstrated the association between gastrointestinal microbiome diversity and tumor-infiltrating lymphocytes (TILs) in breast cancer patients, while Qin et al. (2012) conducted a metagenome-wide association study to identify gut microbial markers for type 2 diabetes (**Table 1**). Overall, this table underscores the scientific impact of these studies in advancing our understanding of the complex interactions between gut microbiota and adaptive immunity in various human diseases, offering potential insights into novel diagnostic and therapeutic strategies.

**Table 1:** Outlines various clinical trials investigating the relationship between gut microbiota and adaptive immunity in a variety of human diseases, from colorectal cancer and rheumatoid arthritis to multiple sclerosis and type 1 diabetes.

Authors (years)	Categories of Study	Gut Microbiota	Study Summary
Cao, Yuan, et al., 2023 (99)	Clinical trial Colorectal Cancer (CRC)	*Fusobacterium nucleatum* (*F. nucleatum*), *Escherichia coli* and *Dasheen mosaic virus*	This study distinct features of the expressed TCRβ repertoires in the peripheral blood of and CRC patients (*n* = 107) and healthy donors (*n* = 30). Collectively, our large-cohort multi-omics data aimed to identify novel biomarkers to inform clinical decision-making in the detection and diagnosis of CRC, which is of possible etiological and diagnostic significance.
Wang, Qi, et al., 2022 (100)	Clinical trial Rheumatoid Arthritis	Actinobacteria Proteobacteria Faecalibacterium, Blautia, Escherichia-Shigella Bifidobacterium	This study investigated the association between intestinal microbiota abundance and diversity and cluster of differentiation (CD)4C T cell subpopulations, cytokine levels, and disease activity in rheumatoid arthritis RA. These results suggest that dysbiosis of certain bacterial lineages and alterations in gut microbiota metabolism leads to changes in the host immune profile that contributes to RA pathogenesis.
Siobh_ (2020) Choileáin, Siobhán Ní, et al., 2020 (101)	Clinical trial Multiple Sclerosis (MS)	Coprococcus, Clostridium, and an unidentified Ruminococcaceae genus	This study investigated the association between the gut microbiomes. and inflammatory T cell subsets in relapsing-remitting MS patients. They show that alpha diversity. inversely correlated with a CXCR3þ Th1 phenotype in MS. These findings indicate the presence of an aberrant autoimmune axis in patients with MS.
Li, Yuan, et al., 2020 (102)	Clinical trial Rheumatoid Arthritis	Clostridium_XlVa,Blautia, Ruminococcus Pelagibacterium, Oxalobacter, ClostridiumXlVb, and ClostridiumXVIII	This study Associations between bacterium and lymphocyte subpopulations as well as cytokines in patients with RA. They show that Gut microbiome of RA patients was clearly different from that of HCs. Abnormal bacteria communities are associated with the altered levels of lymphocyte subpopulation and cytokines, which might be one of the pathogeneses of RA.
Sun, Yang, et al., 2019 (103)	Clinical trial Rheumatoid Arthritis	*Bacteroides Escherichia-Shigella* *Lactobacillus* *Alloprevotella Enterobacter Odoribacter*	This study identifies differences, in the fecal microbiomes of 66 Chinese patients with RA and 60 healthy Chinese controls. These findings suggest that the gut microbiota may contribute to RA development via interactions with the host immune system.
Shi, Jiajie, et al., 2019 (104)	Breast Cancer	*Mycobacterium, Rhodococcus, Catenibacterium, Bulleidia, Anaerofilum, Sneathia, Devosia*	In this study, the gastrointestinal microbiome, and tumor–infiltrating lymphocytes (TILs) were compared in patients with breast cancer (BC). Collectively, the diversity of the gastrointestinal microbiome was associated, with the expression of TILs in patients with BC.
Saresella, Marina, et al., *2017* (105)	Clinical trial Multiple Sclerosis (MS)	*Lachnospiraceae*	multiple sclerosis (MS), has been linked to an alteration of the resident microbial commensal community and of the inter-play between the microbiota and the immune system. Dietary components such as fiber, acting on microbiota composition, could, in principle, result in immune modulation and, thus, could be used to obtain beneficial outcomes for patients. Diet modulates dysbiosis and improves clinical parameters in MS patients by increasing anti-inflammatory circuits. Because *Lachnospiraceae* favor Treg differentiation as well as TGFβ and IL-10 production this effect could be associated with an increase of these bacteria in the microbiota.
Liu, Xiaofei, et al., 2013 (106)	Rheumatoid Arthritis (RA)	*Lactobacillus salivarius, Lactobacillus iners Lactobacillus ruminis Porphyromonas gingivalis Segmented filamentous bacteria (SFB) Lactobacillus bifidus*	The objective of this study was to analyze the human fecal Lactobacillus community and its relationship with rheumatoid arthritis. These results suggest a potential relationship between Lactobacillus communities and the development and progression of rheumatoid arthritis
Qin, Junjie, et al., 2012 (107)	Type 2 diabetes	*Escherichia coli*	They developed a protocol for a metagenome-wide association study (MGWAS) and undertook a two-stage MGWAS based on deep shotgun sequencing of the gut microbial DNA from 345 Chinese individuals
Ng, S. C., et al., 2011 (108)	Clinical trial Crohn’s Disease (CD)	*bifidobacteria, bacteroides-prevotella, C. coccoides-E. rectale, and Faecalibacterium prausnitzii.*	This study relationship between DC function with disease activity and intestinal microbiota in patients with CD. They show that IL-6 production by intestinal DC is increased in CD and correlates with disease activity and CRP. DC function may be influenced by the composition of the commensal microbiota.
Saunders, Karin A., et al., 2007 (109)	Type 1 Diabetes	*Clostridium Bacteroide*	The aim of our study was to examine whether infection with the gastrointestinal helminths Trichinella spiralis or Heligmosomoides polygyrus could inhibit the development of autoimmune diabetes in NOD mice and to analyze the mechanisms involved in protection and the role of Th2 responses. Protection from diabetes was afforded by helminth infection, appeared to inhibit autoimmune diabetes by disrupting pathways leading to the destruction of beta cells, and was mediated by seemingly independent mechanisms depending on the parasite but which may be to be related to the capacity of the host to mount a Th2 response

Each study contributes to understanding of how changes in the composition and function of the gut microbiota can affect the immune system and lead to the development or progression of various diseases.

## 3 Interaction between gut microbiota and T cell function

During the past decade, research has revealed a more intricate relationship between gut microbiota and cellular immunity (27, 28). Adaptive T cells lead defense against immune-mediated inflammatory diseases in cellular immunity, preserving host homeostasis (29). To maintain host immune homeostasis, the microbiota contributes to the activation, polarization, function, and differentiation of T cells (30). The gut microbiota interacts with T cells through antigen-specific recognition or signals via Toll-like and Nod-like receptors (31). Toll-like receptors (TLRs) are highly conserved molecules that enhance immune responses by detecting microbial-associated molecular patterns (MAMPs). While traditionally believed to enhance immunity, recent findings suggest that TLR signaling on T cells can dampen immune responses. Round et al. have shown that the symbiotic factor (PSA, polysaccharide A) of B. fragilis activates TLR2 directly on Foxp3+ regulatory T cells (Tregs), leading to the induction of mucosal tolerance, suppression of anti-bacterial immune responses, and facilitation of colonization in a distinct mucosal environment during homeostasis. These signals mediate cell induction and function, thus ensuring homeostasis in the human immune system (32).

Recent research indicates that microbial molecules and metabolites originating from the gut microbiota may play a role in the maturation of T cells in the thymus, either directly through the presentation of microbial epitopes or indirectly by influencing the selection process. Commensal metabolites like PGN, PSA, DNA, antigens, and SCFA generated in the gut are transported to the thymus via circulation, potentially facilitated by migratory DCs such as pDCs and CX3CR1+ DCs, impacting the development of both conventional and unconventional T cell subsets (33).

### 3.1 Interaction between gut microbiota and T CD4+ cell function

CD4+ T cell differentiation is affected by the makeup and richness of gut microbiota. These CD4+ T cells primarily exist within the lamina propria and lymphoid follicles, predominantly as effector or memory cells. Interactions between lamina propria CD4+ T cells and the gut microbiota, often with epithelial cells or DCs functioning as the intermediaries, are critical for shaping the adaptive immune response in the intestine (33–35).

Microbial metabolites like SCFAs and Polysaccharide A (PSA) are important in regulating the function of CD4+ T cells. They can impact the formation, specialization, and effectiveness of CD4+ T cells, which are essential for the immune system. Research has demonstrated that SCFAs can enhance the formation of Tregs, a subset of CD4+ T cells that play a role in preserving immune tolerance and preventing excessive inflammation.

SCFAs like acetate, propionate, and butyrate naturally inhibit histone deacetylase (HDAC), with butyrate inhibiting up to 80%, propionate up to 60%, and acetate having the lowest inhibition rate. Commensal microorganisms produce butyrate and propionate during starch fermentation, enhancing peripheral Treg cell generation by inhibiting HDAC. In contrast, acetate, lacking significant HDAC inhibitory properties, does not impact Treg cell production. SCFAs indirectly promote Treg cell growth and IL-10 production through interactions with other cell types. For example, butyrate can affect macrophages and dendritic cells (DCs) in a GPR109A-dependent manner, indirectly inducing FoxP3+ T-cells and IL-10 production. More studies have shown that short-chain fatty acids, especially butyrate, also enhance the population of CD8+ memory T cells in the spleen and liver, contributing significantly to the development of an effective secondary antigen-specific response. This mechanism is commonly associated with the upregulation of glycolysis and mitochondrial mass, supporting the longevity and activation of CD8+ memory T cells (36, 37).

An additional compound produced by microbes that is of importance is indole-3-aldehyde (IAld), which is formed through the breakdown of dietary tryptophan by specific bacteria (such as Lactobacillus spp, Clostridia, Bifidobacterium spp, and Peptostreptococcus spp) (38). IAld boosts the generation of interleukin-22 (IL-22) by CD4+ T cells (39). IL-22 plays a role in maintaining the integrity of the intestinal barrier and fostering tissue repair. The balance between different T cell subsets is influenced by signals and metabolites provided by the gut microbiota, shaping CD4+ T cell differentiation significantly. Insights into developing therapeutic strategies, targeting CD4+ T cell-mediated immune disorders, and maintaining immune homeostasis can be gained by understanding these interactions (40).

#### 3.1.1 The regulatory role of the gut microbiota on Th1 and Th2 immune response

The immune system is regulated by the gut microbiota through different mechanisms, including adjusting the balance between Th1 and Th2 immune phenotypes. This balance is crucial for preventing excessive or inappropriate immune responses, maintaining homeostasis, and averting diseases like allergies, autoimmunity, and chronic inflammation. The system of Th1/Th2 generates proinflammatory cytokines that control the development, differentiation, and viability of different types of cells participating in innate and adaptive immunity. Th1 cells predominantly activate cell-mediated immunity and inflammation dependent on phagocytes. Conversely, Th2 cells stimulate the accumulation of eosinophils and responses induced by antibodies, while also inhibiting various functions of phagocytic cells. From a conventional viewpoint, the optimal immune state would be one that equally balances “cellular immunity” (representing Th1 cells) and “humoral immunity” (Th2 cells).

**Figure 1:**
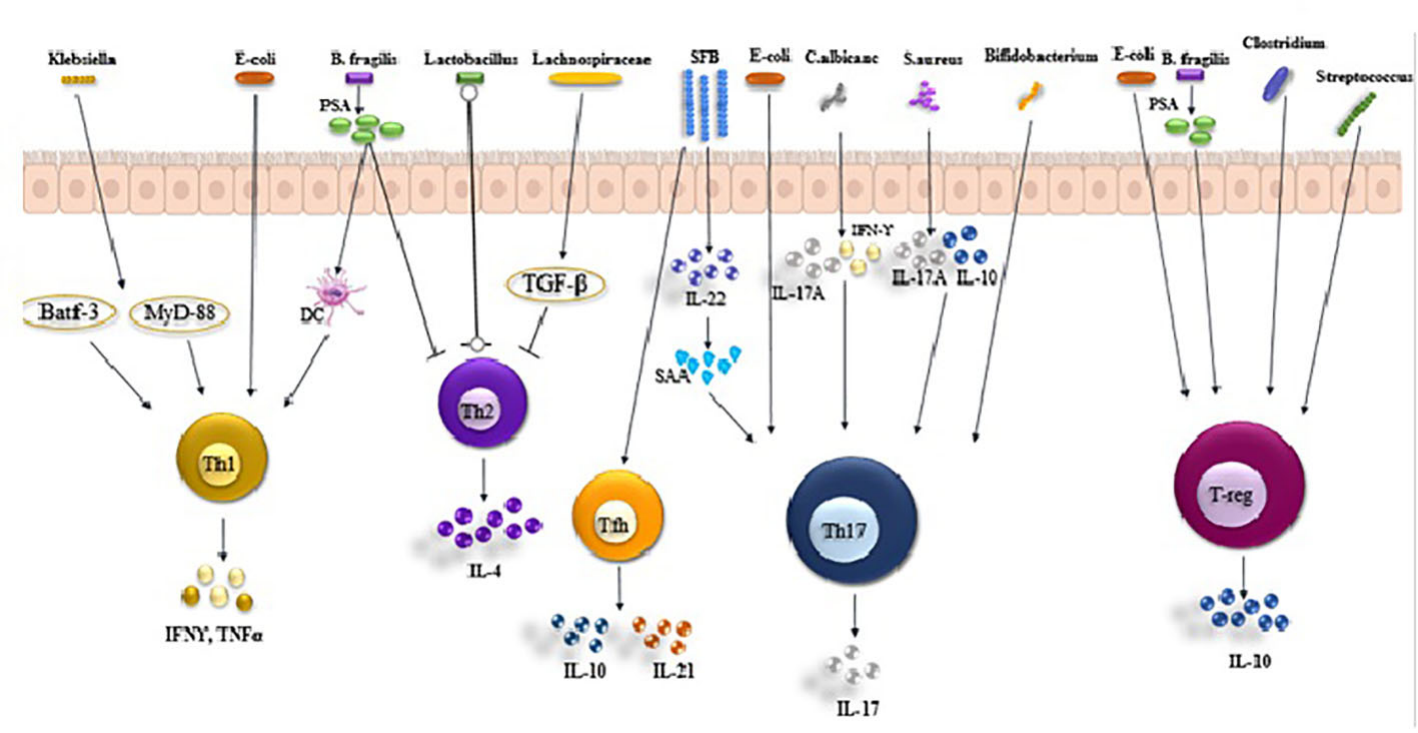
Roles of gut microbiota in Th1, Th17, Treg, and Tfh balance. *Klebsiella*, Ecoli and Bacteroides fragilis(*B. fragilis* produces PSA) activates T helper (Th) 1 cell in the intestinal via antigen presenting cells (APCs) to secrete IFNγ and TNFα. *Lactobacillus* inhibit the production of IL-4 secreted by Th2 cells by APCs. Th17 cells are activated by albicans, *S. aureus, Bifidobacterium*, Ecoli and SFB, secreting IL-17 and causing inflammation. Treg cells are activated by **(B)** *fragilis, Clostridium, Escherichia* and *Streptococcus*, secreting IL-10.

**Figure 2:**
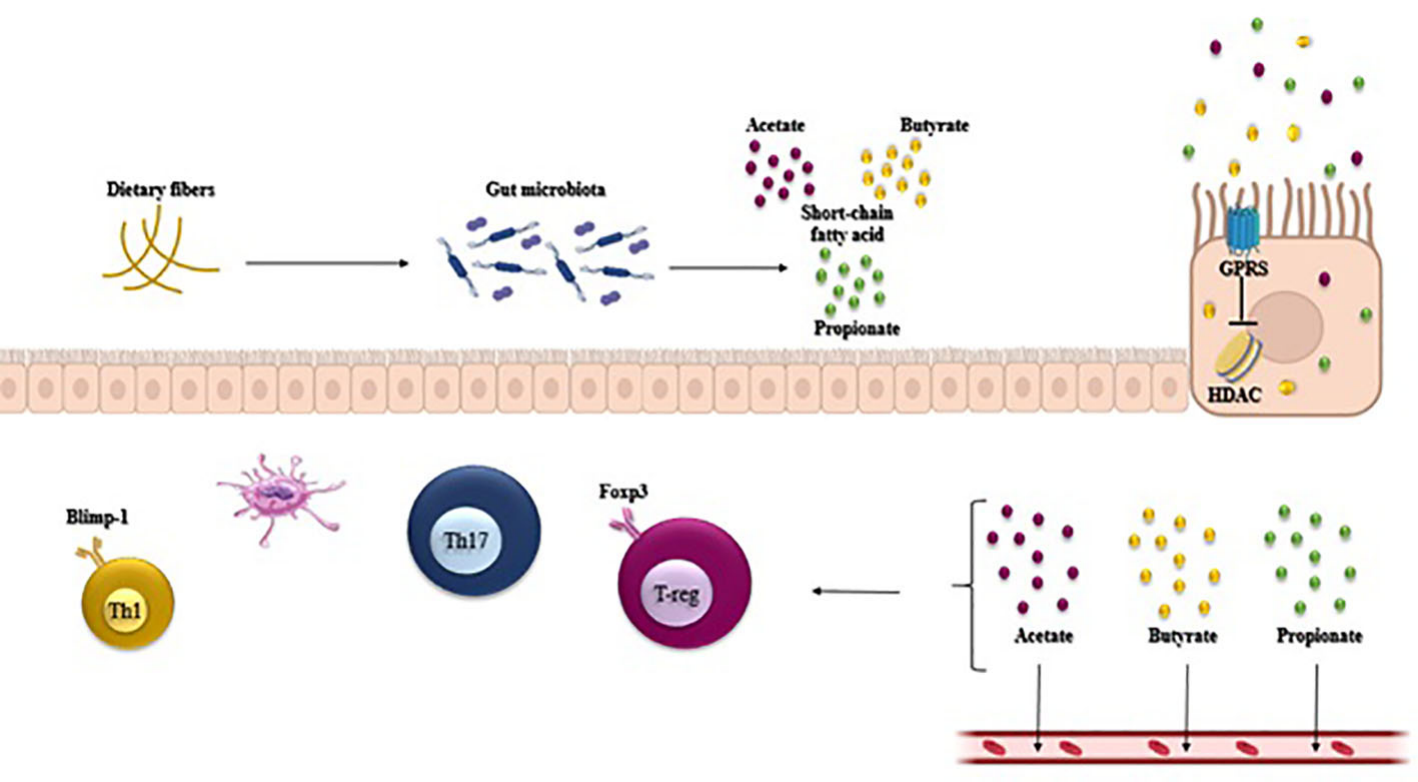
Gut microbiota-derived short-chain fatty acids (SCFAs) such as acetate, propionate, and butyrate regulate host intestinal immune homeostasis. SCFAs regulate the T cell function through G-protein-coupled receptors and through inhibition of histone deacetylase (HDAC), which affects inhibition of nuclear factors (nuclear factor-κB; NF-κB). SCFAs regulate the generation of Th1, Treg, and Th17 in different cytokine environments.

The activation of probiotics leads to the enhancement of downstream signaling molecules, specifically T-bet, STAT-1, STAT-4, IL-2, and IFN-γ, thereby causing Th1-mediated responses (41). Recent research found that when identifying bacterial species from the *Klebsiella genus, like K. aeromobilis and K. pneumoniae*, it activated Th1 cell responses in the gut (42). The induction of Th1 cells by *Klebsiella* requires both the basic leucine zipper ATF-like transcription factor 3 (Batf3) and the MyD88-dependent signaling pathways, both of which contribute to the induction of Th1 immunity (42). Likewise, a strain of *Escherichia coli (E. coli)* found in the small intestine of a patient with Crohn’s disease demonstrated a strong capability to stimulate Th1 cells in germ-free mice (43). Bacteroides fragilis’ polysaccharide A (PSA) is converted into low-molecular-weight carbohydrates using a nitric oxide-mediated process and is presented to T cells by dendritic cells (DCs) via MHC-II. This subsequently triggers Th1 responses (**Figure 1**). In addition, a recent research revealed that Bifidobacterium longum, Bifidobacterium pseudolongum, and a particular combination of strains could also notably enhance the presence of Th1 cells in the small intestinal or colonic lamina propria (44). Multiple studies have demonstrated that probiotic bacteria (*Lactobacillus* (L.), *Plantarum*, and *L. salivarius*) regulate the activity of Th1 cells (45–47). Several research studies have suggested that SCFAs (primarily propionic and butyric acids) activate GPR43 and/or GPR41, leading to the promotion of IL-10 production in microbiota antigen-specific Th1 cells (48, 49). The pathogenic potential of gut microbiota antigen-specific Th1 cells in inducing intestinal inflammation is compromised by SCFAs through the promotion of IL-10. Mechanistically, SCFAs enhance the expression of transcription factor Blimp-1 in Th1 cells, which is contingent on the activation of STAT3 and mTOR (**Figure 2**) (50). The presence of bacteria and viruses in the gut leads to a Th1-, Treg, or Th17-type immune response, whereas the presence of helminths triggers a robust Th2 cell response (51).

Bamias et al. found that both Lactobacillus and B. fragilis strains had a beneficial impact on Th1 activity by inhibiting Th2 activity. In the research conducted by Bamias et al., it was found that the Lactobacillus and B. fragilis strains had a positive impact on Th1 activity by inhibiting Th2 activity (52). The production of transforming growth factor-beta (TGF-β) from DCs, induced by Lachnospiraceae, resulted in the inhibition of Th2 cell differentiation and activity. A recent study suggests that when inflammatory bowel disease (IBD) microbiota is transferred into germ-free mice, it leads to an increase in the amount of intestinal Th17 and Th2 cells, and a decrease in retinoic acid-related orphan receptor γt (RORγt)+ Tregs, in comparison to transferred microbiota from healthy donors (53). Much is still unknown about the impact of specific commensal microbiota on the development of Th1 and Th2 cells, in contrast to what is known about the generation of intestinal Tregs and Th17 cells. Therefore, it is essential to comprehend how these two processes interact with the gut microbiota to create effective treatment approaches for restoring imbalanced conditions linked to disease advancement.

#### 3.1.2 The regulatory role of gut microbiota on Th17 cells

Th17 cells have been extensively studied in microbiota education, serving as the other primary T-cell subset. The CD4+ Th17 subset is distinguished by the expression of the master transcription factor RORγt and the secretion of cytokines, including IL-17F, IL-17A, IL-21, and IL-22. These cells are prevalent among the effector CD4+ T cells in the lamina propria of the intestine. Th17 cells play a crucial role in defending against pathogens, particularly in the context of extracellular bacterial and fungal infections (54).

The majority of Th17 cells in the intestinal lamina propria of mice specifically respond to microbiota antigens, with a strong reactivity towards segmented filamentous bacteria (SFB). These SFBs are beneficial commensal bacteria and are a natural part of the healthy microbiota in various animal species that inhabit the small intestine, especially the terminal ileum (22, 55).

The presence of SFB encourages ILC3s to produce IL-22, which in turn boosts the levels of serum amyloid A (SAAs) in intestinal epithelial cells IECs. When IECs adhere to SFB, they generate reactive oxygen species (ROS) and SAA, which prompt the secretion of IL-1 and IL-23, stimulating the development of Th17 cells. Alongside SFB, other members of the mucosal-associated microbiota such as K. pneumoniae, P. mirabilis, and E. gallinarum are also capable of driving Th17 cell responses (**Figure 1**). The colonization of E.coli produces both mucosal and systemic inflammatory Th17 cells (56), *Bifidobacterium adolescents (* 57), and *Staphylococcus aureus (* 58). *In vivo* models demonstrate the significant impact of bifidobacteria species on maintaining the equilibrium between Th1 and Th2 responses, Th17 cell polarization, and CD8+ cell activation *In vitro* models, all strains of Bifidobacterium have been shown to enhance the production of IL-10 and TNF-. Consequently, bifidobacteria are not only implicated in inflammation but are also linked to the regulation of host immunity. S. aureus strains that colonize produce one or more toxins with superantigenic properties. These superantigenic toxins can activate 10 to 30% of all T cells without requiring processing by antigen-presenting cells (APC). Superantigens directly bind to specific regions of major histocompatibility complex class II molecules on APCs and to the variable region of the β chain (Vβ) of the T-cell receptor (TCR). The findings indicate that superantigenic S. aureus strains are particularly effective in inducing IL-17 production, which is primarily generated from memory T cells.

These microorganisms can mimic the epithelial adherence seen in SFB. However, each microorganism triggers a distinct response in epithelial and Th17 cell transcription and function. For example, Th17 cells stimulated by *C. albicans* produce IL-17A and IFN-γ but not IL-10, while Th17 cells stimulated by *S. aureus* produce IL-17A and IL-10 but not IFN-γ. The precise location and method of Th17 cell induction are not fully understood, but multiple studies have demonstrated that this primarily occurs newly in the small intestine and depends on MHC-II expression by intestinal dendritic cells and macrophages. ILC3s are also implicated in the generation of SFB-specific Th17 cells through the release of IL-22 (**Figure 1**) (59).

The modulation of metabolic activities in the GI tract, such as the secretion of fermentation products like SCFAs (acetate and propionate) by certain bacteria, can impact Th17 cell responses through changes in microbial populations (**Figure 2**).

SCFAs have the potential to engage with specific receptors on immune cells, leading to the reduction or augmentation of cytokine production, thereby either suppressing or boosting inflammatory responses. Additionally, there have been reports of probiotics and dietary fibers containing compounds that help to regulate commensal microbes. This can also aid in controlling the complex interactions between different components that affect Th17 development, ultimately helping to maintain gastrointestinal health (60).

Th17 cells were found to be the primary supporters of antigen-specific IgA generation when exposed to the traditional IgA-boosting adjuvant cholera toxin, as demonstrated by independent research. This suggests that, aside from Tregs, Th17 cells may also play a role in generating IgA against the microbiota. It is worth noting that SFB, a strong promoter of Th17 responses, is also a powerful stimulant of IgA (61).

#### 3.1.3 The role of microbiota in Tregs induction

Recent studies have demonstrated that commensal microbes can induce Tregs in humans, which play a significant role in regulating immune responses to pathogens and other environmental antigens. The natural Tregs that express CD4 and Foxp3 can be found in every body organ. They comprise a high proportion of the T cells of the lamina propria of the intestine. Intestinal Tregs play a critical role in maintaining immune tolerance to dietary antigens and the gut microbiota (62, 63).

Tregs induced by RORγt also have high levels of cytotoxic T-lymphocyte protein 4 (CTLA-4). Gut microbiota stimulates the expression of the transcription factor RORγt. Foxp3+RORγt+ T cells are a crucial subset of effector Tregs in the intestinal immune system and exhibit characteristics of both Tregs and Th17 cells. The close association between Th17 and Tregs makes them exceptional in regulating immune responses and preserving immune homeostasis (64).

Bacterial species have the ability to directly increase the expression of the transcription factor FoxP3, leading to enhanced differentiation into mature Tregs, through the presence of specific microbial components or metabolites referred to as “microbial associated molecular patterns” (MAMPs).

Numerous studies recently found that various members of the microbiota can trigger the induction of Tregs in both the small intestine and lamina propria. The microbiota comprises specific endogenous bacteria (*Clostridium clusters IV, XIVa, and XVIII*), bacterial products (*B. fragilis polysaccharide)*, or bacterial byproducts (such as SCFAs including acetate, butyrate, and propionate), as well as other bacteria like *Escherichia*, *Akkermansia*, *Lactobacillus*, and *Streptococcus* strains (**Figure 1**) (65–67).

The epithelial cells can be prompted by SCFAs to produce TGF-β, thereby aiding in the initiation of peripheral Tregs (14). Butyrate, a significant SCFA in the intestinal tract, is derived from Clostridium clusters IV and XIVa. Butyrate encourages CD103+ DCs to generate elevated levels of TGF-β, which attaches to GPCRs on the DCs (GPR109A) and CD4+ T cells (GPR43) to facilitate Treg differentiation (68). Moreover, butyrate has the ability to block histone deacetylase (HDAC) and encourage the acetylation of histone H3 in the enhancer of Foxp3, leading to the conversion of naive CD4+ T cells into peripheral Tregs (**Figure 2**). Retinoic acid and dietary fiber serve as co-metabolic factors that contribute to the expansion of these CD103+ DC–Treg interactions (69). PSA, which is generated by various Bacteroides species such as Bacteroides fragilis, stimulates Tregs and IL-10-mediated anti-inflammatory responses from T cells and DCs. PSA operates as a symbiosis factor by activating Toll-like receptors 2 (TLR2) and can also reverse Th1- and Th17-polarized lamina propria cells as well as enhance Treg secretion of IL-10 (32, 67).

Several research studies have demonstrated that Tregs play a role in promoting the survival of antigen-specific IgA+ B cells, providing tolerance to commensal bacteria. Depletion of Tregs using anti-CD25 led to the reduction of intestinal IgA B cells (70). Additionally, experiments involving adoptive transfer models revealed that the absence of peripheral Treg (pTreg) induction for certain microbiota antigens resulted in increased susceptibility to intestinal inflammation and the differentiation of T effector cells. Nonetheless, Tregs can exert their suppressive function by secreting anti-inflammatory cytokines such as transforming growth factor-β and IL-10, as well as by inducing IgA directly or through the facilitation of Tfh cells and T follicular regulatory cells, thereby maintaining gut homeostasis (71).

In conclusion, evidence suggests that alterations in the microbial composition resulting from dysbiosis or antibiotic treatments can result in an imbalance between effector subsets driven by the loss of induction pathways for protective cell types such as CD4+CD25+Foxp3+Tregs. Therefore, it is clear why therapeutic strategies attempting to modulate the host immunity must consider the contributions of its associated microbiome when manipulating this delicate system.

#### 3.1.4 The regulatory role of the gut microbiota on Tfhs

The presence of microbiota impacts the number and function of Tfh cells. Conversely, Tfh cells have the ability to influence the microbiota. Tfh plays a crucial role in activating B cells, switching isotypes, and forming germinal centers (72). Eliminating Tfh cells leads to decreased levels of Peyer’s patches (PPs), IgG1, and B cells in germinal centers (GC) (73). Thus, the activity of Tfh cells is essential for fostering a varied microbiota community in the intestinal tract.

The production of IL-21 by Tfh cells in PPs is responsible for driving the GC reaction and high-affinity sIgA production in the small intestine. There is a notable decrease in anaerobic bacteria in the gut of Programmed cell death-1-deficient mice, which lack Tfh cells. Additionally, Tfh cells have the ability to detect bacterial ATP through the P2X7 receptor and subsequently influence the composition of the gut microbiota (74).

The differentiation of Tfh cells is guided by *Akkermansia muciniphila* or SFB, which in turn assists in the curation of microbiota composition through IgA and IgG1. This promotes the general diversity and stability of the microbiota. In the Peyer’s patches, SFB triggers the differentiation of Tfh cells by blocking the IL-2 signaling pathway in CD4 T cells (74, 75).

Furthermore, research has indicated that dysregulation of specific microbial species may lead to alterations in normal immune responses and even chronic inflammatory disorders, such as allergies and autoimmunity. Thus, understanding how our gut microbes interact with Tfh could provide new insights into the pathogenesis and treatment of these diseases.

### 3.2 Interaction between the gut microbiota and T CD8+ cell function

Critical cells for protecting the body against intracellular pathogens such as viruses and bacteria, as well as for monitoring the immune system for tumors, are CD8+ T cells. Studies have shown that microbial antigens and metabolites can interact with antigen-presenting cells in the gut-associated lymphoid tissue, leading to the priming and activation of antigen-specific CD8+ T cells. In addition, specific microbial metabolites, such as SCFAs, have been shown to directly influence CD8 + T cell responses (76).

Nastasi et al. demonstrated that butyrate and propionate, the two primary short-chain fatty acids derived from *Lachnospiraceae* species, have been shown to decrease the production of IL-12 induced by antigen-presenting cells (APC). This action inhibits the activation of CD8+ T cells and the secretion of IFNγ. Additionally, butyrate directly stimulates cytotoxic T lymphocyte (CTL) cells through alternative pathways, leading to an increase in the secretion of IFNγ and granzyme B (GzmB) (77). Sivan et al. showed that probiotic strains like Bifidobacterium and Mobilicoccus massiliensis stimulate CD8+ T cells and boost the production of IFNγ and TNFα, thereby intensifying the anti-tumor CD8+ T cell response (78).

Another study showed that a consortium composed of 4 non-bacterial species acting as effectors and 7 other bacteria acting as supporting elements results in the accumulation of IFNγ+ CD8 T cells in the intestinal lamina propria, significantly affecting the immune system’s function (79).

In this study, Tanoue et al. identified 11 human-associated bacterial strains that collectively demonstrated effectiveness in stimulating IFNγ+ CD8 T cells, enhancing resistance to the intracellular pathogen Listeria, and inhibiting tumor growth in conjunction with immune checkpoint inhibitors (ICIs). These isolated strains exhibit significant biotherapeutic potential and hold promise for widespread application in enhancing cancer and infectious disease treatments (80)

Moreover, the gut microbiota can indirectly influence the CD8+ T cell function by modulating the overall immune environment in the gut. Dysbiosis or alterations in the gut microbial composition have been associated with impaired CD8+ T cell responses and increased susceptibility to infections and inflammatory diseases (81).

## 4 Interaction of the gut microbiota and B-cells

Recent studies have shown that the dynamic interactions between B-cells and gut microbiota are critical for immune system functions, which include protecting humans from infectious diseases. B-cells play a crucial role in intestinal homeostasis by mediating adaptive immunity against pathogens and tolerating commensal bacteria through IgA production. The reciprocal relationship between B cells and the microbial environment is regulated by various signaling pathways involving TLR stimulation, cytokines, immunoglobulins, or other molecules. Recent evidence suggests that changes in the composition of bacterial species within the gut microbiota may lead to alterations in B-cell development and function. This can increase susceptibility to infections or even autoimmune disease development. Therefore, understanding these interactions is essential for elucidating our knowledge about human health and disease prevention since disrupting these interactions can profoundly impact host physiology (82, 83).

### 4.1 The regulatory role of Gut- microbiota on IgA

IgA plays a critical role in gut microbiota interactions, acting as the first line of defense against pathogens and maintaining homeostasis within the gastrointestinal tract. IgA is produced by B-cells in response to microbial products, such as lipopolysaccharides or peptidoglycans from Gram-positive bacteria. The antibody binds to these molecules on the bacterial surface and tags them for degradation via phagocytosis or other mechanisms (70). Furthermore, IgA can be used to recognize commensal microorganisms and prevent their adherence to epithelial surfaces or invasion of mucosal tissues. In return, some gut microbes produce molecules that bind to IgA antibodies, promoting their survival within the intestines; this also aids in developing immune tolerance towards harmless bacteria present in our body (commensalism). As such, IgA is an essential regulator of host-microbiome interactions, and its dysregulation can result in pathological conditions such as IBD or infectious diarrhea (84).

**Figure 3:**
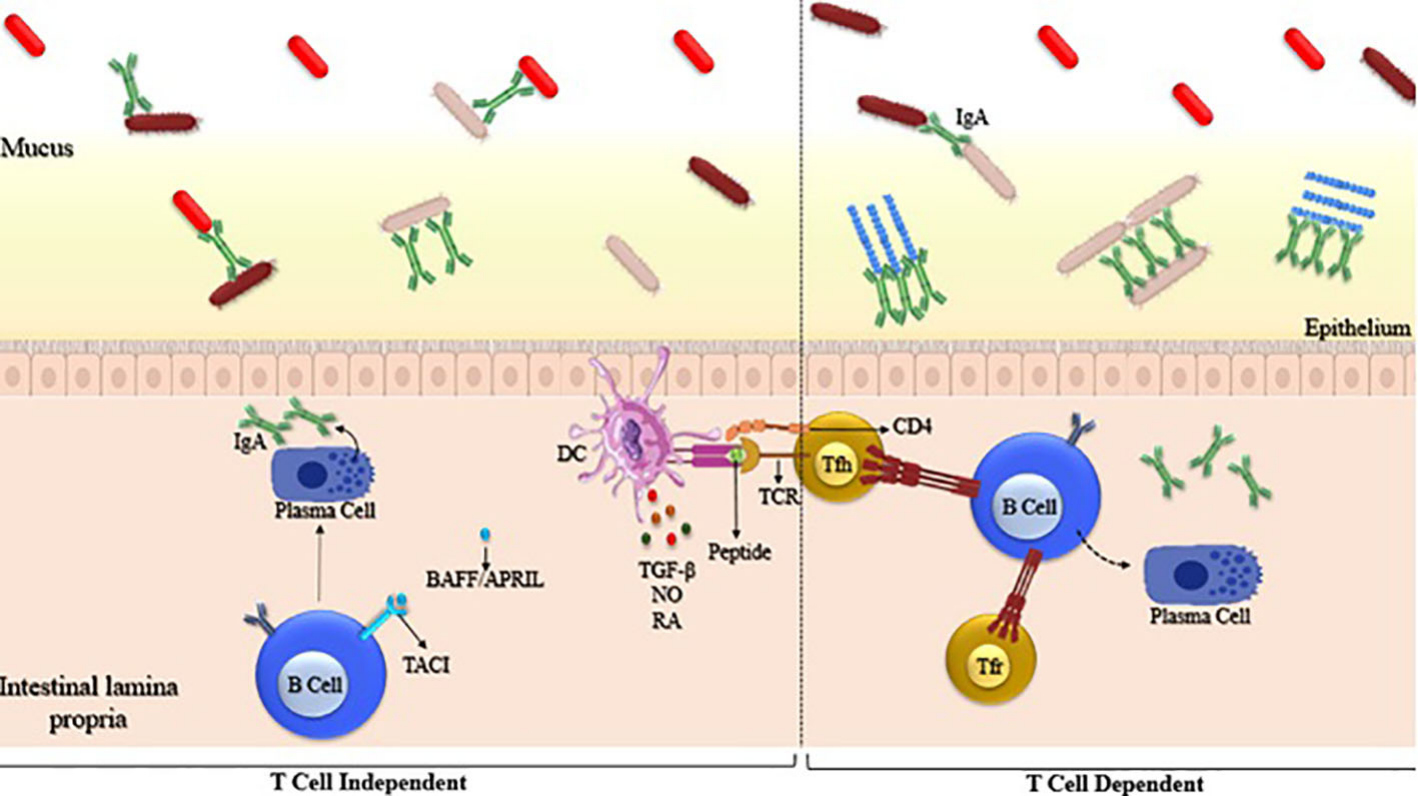
Generation of microbiota-reactive IgA through T cell-independent and T cell-dependent pathways. Left panel: T cell-independent IgA is generated by B cells that are stimulated outside of Peyer’s patches and in the absence of T cells. Right panel: T cell-dependent IgA is generated by B cells after interaction with activated T follicular helper (TFH) cells within Peyer’s patches. Examples of such microbes include segmented filamentous bacteria (SFB) and Mucispirillum. SFB is known to strongly induce the formation of germinal centers (GC) in the Peyer’s patches (PP), which are specialized lymphoid tissues in the intestine.

IgA production can be stimulated by both T-dependent and T-independent pathways, with the primary location for T-dependent IgA production with PP (85) T-dependent IgA appears to be generated in response to microbes closely linked to the intestinal epithelium, such as SFB and *Mucispirillum.* SFB is known for strongly promoting the formation of germinal centers in Peyer’s patches, as well as supporting the development of tertiary lymphoid structures, both of which contribute to IgA production. The production of IgA within PP is under the regulation of both Tfh and T follicular regulatory (Tfr) cells (**Figure 3**).

Even though IgA is the predominant antibody found in the gut, IgM and IgG are also detectable. B cells locally generate IgM and IgA, and class-switch recombination from IgM to IgA is facilitated by PPs and isolated lymphoid follicles. T-independent class switch and IgA induction mechanisms have been documented to involve cytokines that promote class switching (e.g., BAFF, APRIL), dendritic cells, and innate lymphoid cells (86).

The most prevalent antibody found in the bloodstream is IgG, which can also enter the gut through a neonatal Fc receptor. While IgA and IgM are generated in reaction to luminal microbial epitopes that dendritic cells sample, it is believed that IgG production requires antigens to cross the barrier, meaning that IgG is not continuously produced in response to common gut antigens. In the intestine, IgA, IgM, and IgG are antibodies capable of coating different types of bacteria (87).

A reduced proportion of the gut microbiota is coated by IgG. Research has demonstrated that IgG is not continually produced in the gut, as it is primarily triggered by antigens found in pathogens like Haemophilus that can penetrate the intestinal barrier. Conversely, IgA and IgM coat similar members of the microbiota found in the lumen. However, the concentration of IgM is nearly 100 times lower than that of IgA. Currently, only a few studies have delved into the role of IgG and IgM antibodies in host-microbiota symbiosis (88).

## 5 Interaction of the gut microbiota and memory T cells

The microbiota plays a crucial role in the function and development of effector and memory T cells. Memory T cells encompass various subtypes, including central memory T cells (Tcm cells), effector memory T cells (Tem cells), and Tissue Resident memory (Trm) cells. Trms primarily reside in tissues and are essential for rapid protection against invading viruses and bacteria. Since tissues are significantly influenced by the microbiota, it follows that Trms are also impacted by the microbiota present in barrier sites. Research indicates that microbial metabolites directly and indirectly affect the differentiation, function, and survival of Trm cells in tissues (89).

The microbiota primarily exerts its influence through the production of microbial metabolites. Short-chain fatty acids (SCFAs) play a role in promoting the formation of long-term memory. For instance, butyrate enhances fatty acid uptake and oxidative phosphorylation in CD8+ memory T cells, supporting the survival of memory cells. Acetate is directly taken up by CD8 T cells, boosting glycolytic activity by increasing acetyl-CoA levels, thereby expanding the CD8+ memory T cell population (90). SCFAs also stimulate the production of IL-10, crucial for the development of memory CD8 T cells (91). Additionally, retinoic acid (RA) is necessary for the expression of the chemokine receptor CCR9 and integrin α4β7, essential surface proteins for T cell trafficking and the development of tissue-resident memory T cells in the small and large intestine (92). The microbiota also modifies bile acids produced by the liver to aid in fat digestion, with bile acids directly influencing effector/memory T cell homeostasis in the gut. Furthermore, the microbiota plays a role in the production of cytokines that support tissue-resident memory T cell survival, such as IL-7 and IL-15 (93).

## 6 Interaction of the gut microbiota and vaccine responses

The host’s immune system influences the response to vaccination. Studies indicates that the gut microbiota composition plays a crucial role in controlling immune responses, potentially affecting how individuals respond to vaccination. Additionally, factors such as age, gender, environment, diet, chronic infections, chemotherapy, antibiotic treatment, and probiotic use influence the composition of the microbiota community.

The effect of gut microbiota composition on vaccine immunogenicity is thought to involve cross-reactive epitopes between by microbes and vaccine antigens, as well as the influence of microbial metabolites like short-chain fatty acids (SCFAs) on B cell responses. Additionally, specific microbes are suggested to provide natural adjuvants in this mechanism. For instance, short-chain fatty acids (acetate, propionate, and butyrate) enhance B cell metabolism and control gene expression to support the differentiation of B cells into cells that produce antibodies (94).

Clinical trials have been conducted to investigate the relationship between gut microbiota and vaccine response in humans of different ages in countries with varying income levels. The potential impact of gut microbiota on vaccines such as OPV, BCG, TT, HBV, and RVV has been examined. These results support the correlation between microbiome composition and vaccine immunogenicity (95).

Borgognone et al., showed that the presence of the Roseburia genus, known for producing butyrate, is directly linked to the levels of interleukin (IL)-27 in circulation and the T cell response to the HIV cell vaccine (96).

Huda and colleagues investigated the potential role of the microbiota in the immune response to injectable vaccination in Bangladeshi infants. Actinobacteria (Bifidobacterium) were predominant in infants. A positive correlation was observed between Bifidobacterium and certain adaptive immune responses, such as CD4+ and CD8+ T cells and specific IgG responses to TT, OPV, BCG and Hepatitis B (HBV) vaccines. Their findings indicated that enhancing the presence of intestinal Bifidobacteria and reducing dysbiosis in infancy can enhance vaccine responsiveness (97). Lynn et al. demonstrated that dysbiosis induced by antibiotics results in a compromised antibody response to various vaccines, such as Meningococcal serogroup B and C vaccines, the 13-valent pneumococcal conjugate vaccine; the hexavalent combination vaccine, BCG and TIV OPV vaccine administered early in the life of mice (98).

## 7 Conclusion

Gut microbiotas have a crucial role in developing and maintaining adaptive immunity. They can induce T-cell and B-cell responses, providing an adaptive mechanism of protection against pathogens. The specific type of bacteria present in the gut influences the strength and specificity of these responses and their ability to recognize antigens encountered during infection or vaccination. This regulation may be responsible for proper function during the host defense against pathogen invasion or immune response to foreign substances such as food allergens or drugs. Furthermore, gut microbiota is essential for the development and maturation of Tregs, which balances homeostasis between various immune cell types, and ensures that appropriate immune responses are generated while eliminating unnecessary activation that might lead to autoimmune diseases such as allergies or asthma. In conclusion, the gut microbiota could play a critical role in regulating adaptive immunity by controlling inflammation levels and directing pro/anti-inflammatory pathways depending on environmental cues within its environment. This ultimately leads to efficient protection from potential pathogens while avoiding overactive reactions towards harmless molecules like food components.
